# Post-quake call for action: developing core competencies matrix for Syrian health workers in emergency time

**DOI:** 10.1186/s13031-023-00567-0

**Published:** 2024-01-05

**Authors:** Hani Saeed, Sulaf Hamid, Imad Zoukar, Adel Khiami, Lama Al Hawat, Mohammed Khoja, Hossam Khawatmy, Hani Abdalnour, Mayssoon Dashash

**Affiliations:** 1 Medical Education Program, Syrian Virtual University, Damascus, Syria; 2 Faculty of Nursing, Latakia, Syria; 3 Pediatric Dentistry Department, Qasyoun Private University for Science and Technology, Damascus, Syria; 4 Department of Pediatrics, Damascus Hospital, Damascus, Syria; 5 Research Department, Demonstration Training and Research Oral Health Center (DTROHC), Ministry of Education, Damascus, Syria; 6 Department of Neurosurgery, Faculty of Medicine, Damascus University, Damascus, Syria; 7 Department of Pediatric Dentistry, Faculty of Dentistry, Damascus University, Damascus, Syria

**Keywords:** Competencies, Syria, Health workers, Emergency, Earthquake, Disaster

## Abstract

**Background:**

The recent earthquake in Syria has caused widespread devastation, leading to extensive damage and loss of life. Considering the diverse range of disasters and conflicts that have affected Syrian society, health workers must possess essential competencies to effectively manage various types of disasters, including earthquakes. Therefore, this study was undertaken to identify the specific competencies required by Syrian health workers to respond efficiently and effectively to earthquakes.

**Methods:**

An exploratory qualitative study was conducted at the Medical Education Program MEP of the Syrian Virtual University SVU. Nine members of the research team of the MEP, who represent various health specializations in medicine, dentistry, nursing, and pharmacy, accepted to take part in this study. Among these, three members have been actively involved in providing health care in hospitals and the field during the Syrian earthquake on 6 February 2023. The Delphi process was adopted to identify competencies. Health workers involved in earthquake response were categorized into nine groups including medical doctors, dentists, pharmacists, nurses, psychological support professionals, medical students, allied healthcare professionals, on-site disaster teams, and managers. The final list was accepted if it achieved more than 80% agreement among the participants in the first, second, and final rounds.

**Results:**

The study identified 74 competencies (12 knowledge items, 35 skills, and 27 attitudes) essential for health workers to respond effectively to earthquakes. They are categorized into five domains: "Preparing the team for the rescue process during and, after earthquakes, Implementation of the rescue process, Education and psychological support, Research, and development".

**Conclusion:**

A list of earthquake competencies was identified for health workers. It is hoped that this list will enhance a country’s resilience and will enable decision–makers to support health workers in acquiring these competencies within a very strained health system in Syria and other countries.

## Introduction

Syrian people woke up to a new tragedy on February 6 when a magnitude 7.8 earthquake struck the border between Syria and Turkey, causing significant damage and loss of life [1]. The latest reports estimate that over 1.5 million people have been affected [1]. The country had already suffered from almost 12 years of crisis, which has led to poor medical infrastructure, and equipment, and consequences that may lead to inadequate response and preparedness when a massive earthquake occurs due to shortage and destruction of facilities and resources [2].

Some health workers can be part of the first responders to earthquakes. The major groups of first responders include emergency medical teams, firefighters, police officers, and search and rescue teams.

Health workers are all people engaged in work actions whose primary intent is to improve health, including doctors, nurses, midwives, public health professionals, and laboratory technicians [3]. The term also includes health management and support workers such as cleaners, drivers, hospital administrators, district health managers, and social workers [3]. Therefore, they should have essential competencies to respond appropriately to the disaster.

Health professionals diagnose, treat, and prevent human illness, injury, and other physical and mental impairments. They also conduct research and improve or develop concepts, theories, and operational methods to advance evidence-based health care. Their duties may include the supervision of other health workers [4]. Previously, emergency response leaders and experienced disaster workers have provided evidence that not all people function well in disasters [5]. Response to an earthquake disaster requires health workers to be equipped with essential competencies not typically used in daily medical practice [6]. They should have a robust foundation in emergency medicine and skills to perform basic medical procedures in challenging and resource-limited environments [7, 8].

In addition, health workers responding to disasters should work effectively as part of a multidisciplinary team, collaborating with other first responders, including search and rescue teams [9]. Search and Rescue team is a specialized group of individuals with appropriate equipment, skills, and training during the disaster response phase. They search for victims trapped under debris and provide initial medical treatment to victims during extrication operations [9]. Moreover, each member should be equipped and trained to be self-sufficient for the first 72 h [10]. An effective disaster health workforce should consist of individuals and groups with diverse competencies and consistently perform their assigned roles well [5].

Moreover, natural disasters including earthquakes cause significant psychological and social suffering to the affected population, making protecting and improving people's mental and psychosocial well-being a priority in emergencies [11]. Therefore, one of the urgent responses during a disaster is to provide medical and psychological first aid (PFA) to injured people to ensure continuous access to essential psychosocial support [1, 11].

Several efforts have been performed to identify disaster medicine competencies to advance disaster preparedness internationally [7, 12–14]. For instance, Su et al. [14] have identified skills that nurses should possess to respond effectively to five types of natural disasters including earthquakes, typhoons, tsunamis, marine disasters, infectious diseases, and three man-made disasters. Five types of professional skills have been identified including triage, observation, monitoring techniques, psychological care, and communication skills. Researchers have indicated that different disasters require different specific skills [14].

Additionally, previous work in Yemen has indicated that the current knowledge, attitude, and training in emergency and disaster preparedness among Yemeni health professionals are inadequate and suggested the implementation of teaching programs [15].

In Syria, there is no study, which identifies the essential competencies required for health workers to respond effectively to the earthquake. Therefore, this study aimed to identify competencies needed for Syrian health workers to respond to earthquakes.

## Methods

An ad hoc research team in the Medical Education Program MEP at the Syrian Virtual University SVU was established by the program director Mayssoon Dashash MD to respond to health and educational challenges during the Syrian crisis [16–20], the COVID-19 pandemic [21, 22], and the earthquake. The sudden earthquake affecting Syrian society addressed the gap of knowledge and skills of some health workers in responding to the earthquake. The director MD called for research that can fill the gap and identify essential competencies required for responding to this event. The research question was raised by MD as "What competencies are needed for health workers to respond to disasters effectively, more specifically following earthquakes?".

Ethical approval was obtained from the research committee of the SVU, No. 490/0, dated 20/03/2023, to undertake an exploratory qualitative study.

To promote participation, a one-week window was provided, and a WhatsApp group was formed to invite all members of the research team in the MEP, who were located in the area affected by the Syrian earthquake to actively engage in this study. Nine research members, who represent various health specializations in medicine, dentistry, nursing, and pharmacy, participated in the study. Among these, three participants have been actively engaged in providing health care in hospitals and the field during the Syrian earthquake on February 6th, 2023.

Based on previous work [21], the Delphi process was adopted to develop consensus on the essential competencies required from health workers and supportive team in response to disasters specifically, earthquakes. The Delphi process consisted of four stages: preparation, first round, second round, and third round.

During the preparation stage, the research team conducted an extensive literature review on emergency care, earthquake competencies, and disaster management to create a preliminary list of competencies.

To obtain the relevant resources for the literature review, a framework such as Participant, Concept, and Context PCC, was used to form a research question 'What are the competencies (Context) required for health workers (P) to respond to the earthquake"(C). The type of population was health workers who are engaged in improving the health of injured and affected people in the earthquake field. The context was the competencies including knowledge, skills, and attitude required. The concept was the earthquake. It was important to pay attention to the aim of the study to identify the essential competencies required to approach affected people by the earthquake. The keywords used in the search were" earthquake", health workers and competency. Resources were obtained from Scopus and PubMed. All publications were tested according to inclusion and exclusion criteria. The search was limited to English peer-reviewed articles. All peer-reviewed journal articles and related references, which include studies that explored the competencies of health workers and used quantitative, qualitative, or mixed methods, were also searched. Publications that were not original research including letters to editors, poster or conference proceedings, and book chapters were excluded. Keywords and alternative words were identified and combined using OR/AND. The search yielded 41 papers including 36 in PubMed, and 5 in Scopus (Table 1). Based on the inclusion criteria, the titles of retrieved articles were screened by MD, SH and EZ. All references were managed by reference manager software (Mendeley, Elsevier). In addition, the reference list of each article was also scanned to identify any article that might fit the identified inclusion criteria. The quality of studies was assessed by taking into consideration the clarity of the study aim, the appropriateness of the methodology applied, the data analysis, and the clarity of findings [23].

**Table 1:** Search strategy

Database	Search strategy
Scopus: 5	TITLE-ABS-KEY (healthcare AND worker) AND TITLE-ABS-KEY ( Earthquake) AND TITLE-ABS-KEY (competency) Scopus—Document search results |. Retrieved February 17, 2023
PubMed: 36	Search: Search: ((Earthquake) AND (healthcare worker)) AND (competency) Sort by: Most Recent ("earthquake s"[All Fields] OR "earthquakes"[MeSH Terms] OR "earthquakes"[All Fields] OR "earthquake"[All Fields]) AND ("health personnel"[MeSH Terms] OR ("health"[All Fields] AND "personnel"[All Fields]) OR "health personnel"[All Fields] OR ("healthcare"[All Fields] AND "worker"[All Fields]) OR "healthcare worker"[All Fields]) AND ("compete"[All Fields] OR "competed"[All Fields] OR "competences"[All Fields] OR "competencies"[All Fields] OR "competently"[All Fields] OR "competent"[All Fields] OR "competes"[All Fields] OR "competing"[All Fields] OR "mental competency"[MeSH Terms] OR ("mental"[All Fields] AND "competency"[All Fields]) OR "mental competency"[All Fields] OR "competence"[All Fields] OR "competency"[All Fields] OR "competent"[All Fields]), English and human Retrieved February 17, 2023

The majority of studies reported nursing skills required across natural and man-made disasters rather than other health professionals. Limited publications reported the psychosocial support skills of health professionals to improve the mental health of earthquake victims [24, 25].

In the primary list of competencies, health workers involved in earthquake response were categorized into nine groups: medical doctors, dentists, pharmacists, nurses, psychological support professionals, medical students, allied healthcare professionals, on-site disaster teams, and managers.

The research participants collaboratively prepared a preliminary list (pilot matrix) of competencies based on the literature review. After modification, refinement, and duplication removal, these competencies were categorized into knowledge, skills, and attitude domains.

Knowledge can be defined as the capacity to acquire, retain, and use information. It requires intellect and the power of sense to acquire the concept and distinguish between right and wrong [26]. Skill can be defined as the application of rules and knowledge that leads to action, which should be guided by a special ethical code such as balancing between anticipated benefit and potential risk [26].

Attitude refers to preferences to react to certain situations in a certain way. It can be defined as the interpretation of events according to certain predispositions and organizing opinions into coherent and interrelated structures such as values and a description of ethical actions [26].

Subsequently, participants engaged in six online meetings, each lasting two hours, to discuss and evaluate the importance of each competency in the pilot matrix for each of the nine groups. WhatsApp application was selected as a tool for communication and organizing meetings through either messages or voice notes.

A coding system was employed to evaluate the competencies, using symbols to represent different levels of competency: “ +  +  + ” for competent, “ +  + ” for knowledgeable, “ + ” for familiar, and “−” for non-essential competencies. This process utilized a matrix table with color coding.

At the end of the discussions, the pilot matrix was transformed into a structured matrix and proceeded to the first round of the Delphi process. Competencies were categorized into domains and subdomains.

During the meeting sessions, participants cooperated and shared their perspectives and experiences on the competencies required for health workers to respond effectively to an earthquake. Each participant contributed constructively and provided insightful thoughts during the dynamic discussion.

In the first round of the Delphi process, all nine members individually voted on each competency in the structured matrix. Each participant responded to his/her professional group and provided an opinion towards the competencies items of the other group. Only competencies that achieved more than 80% agreement progressed to the second round. Participants had the flexibility to add new competencies and change their responses during this round.

Following the first round, the structured matrix underwent a review process. Modifications and refinements were made by the research team based on the feedback received from three participants working in the earthquake field who have been providing emergency health care to affected people in the field and hospital. Additionally, new suggested competencies were included, and redundancies were removed to create a better-organized and more practical list of essential competencies.

In the second round of the Delphi process, participants were invited by the team leader MD to vote again on the revised version of the structured matrix. They were asked to re-score the importance of each competency, with the option to change their responses if desired. Competencies that achieved more than 80% agreement progressed to the final round.

In the third round of the Delphi process, participants were invited for the last time to provide their agreement or disagreement with the final list of competencies as a whole. The final list was accepted if it achieved more than 80% agreement among the participants.

## Results

The preliminary list of competencies consists of 133 items divided into three categories: knowledge with 31 items, skills with 46 items, and attitude with 56 items. These competencies are further classified into six domains:

1. Fourteen items (10.5%) related to *"Team readiness for rescue operation*" (six knowledge, five skills, and three attitude items).
2. Nine items (6.8%) related to *"Preparation for rescue*" (4 knowledge, 4 skills, and 1 attitude item).
3. Twenty-eight items (21%) related to *"Execute rescue operation"* (three knowledge, 14 skills, and 11 attitude items).
4. Sixteen items (12%) related to "*Psychological support*" (six knowledge, seven skills, and three attitude items).
5. Sixteen items (12%) related to "*Research, development, and education*" (eight knowledge, six skills, and two attitude items).
6. Fifty items (37.6%) related to "*Leadership competencies*" (four knowledge, 10 skills, and 36 attitude items).

In the first round of the Delphi process, all nine participants provided their responses and evaluated the structured matrix, resulting in a response rate of 100%. Out of the 133 competencies initially considered, only 84 (63.2%) competencies received an agreement of 80% or higher and advanced to the second round.

These competencies were reorganized into five domains and distributed across three categories: knowledge (19 items), skills (49 items), and attitude (16 items).

Moving to the second round of the Delphi process, all nine participants (maintaining the 100% response rate) reviewed and assessed the revised structured matrix. Only 74 competencies out of the initial 84 achieved an agreement of 80% or higher and progressed to the third round.

In the third round of the Delphi process, all nine participants unanimously approved the final list of competencies as a whole, indicating a complete consensus on the selection.

The final list of competencies comprises 74 essential elements, including 12 knowledge areas, 35 skills, and 27 attitudes. These competencies have been the most crucial for health workers to be able to respond to earthquakes effectively. They are categorized into five domains, as shown in Table 2:

1. "Preparing the team for the rescue process during earthquakes" comprises fifteen items (20.3%), including two knowledge, seven skills, and six attitude items.
2. "Preparing the team for the rescue process after earthquakes" consists of fifteen items (20.3%), including 3 knowledge, 5 skills, and 7 attitude items.
3. "Implementation of the rescue process" encompasses twenty-six items (35.1%), including two knowledge, 14 skills, and 10 attitude items.
4. "Education and psychological support" includes nine items (12.2%), consisting of three knowledge, four skills, and two attitude items.
5. "Research and development" covers nine items (12.2%), including two knowledge, five skills, and two attitude items.

**Table 2:**
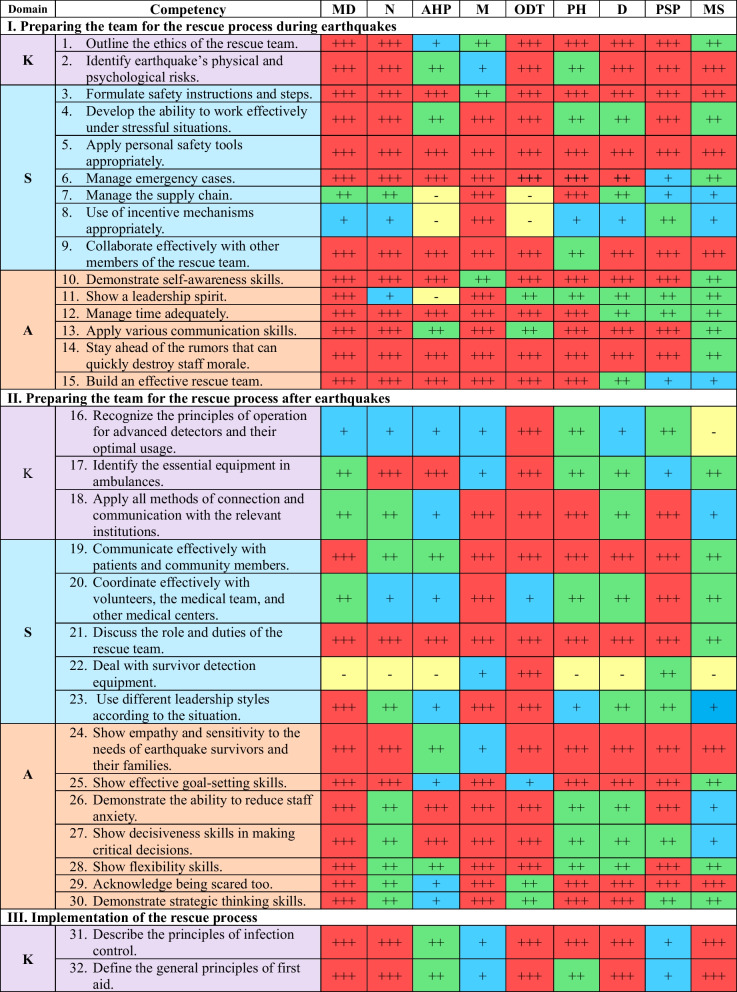

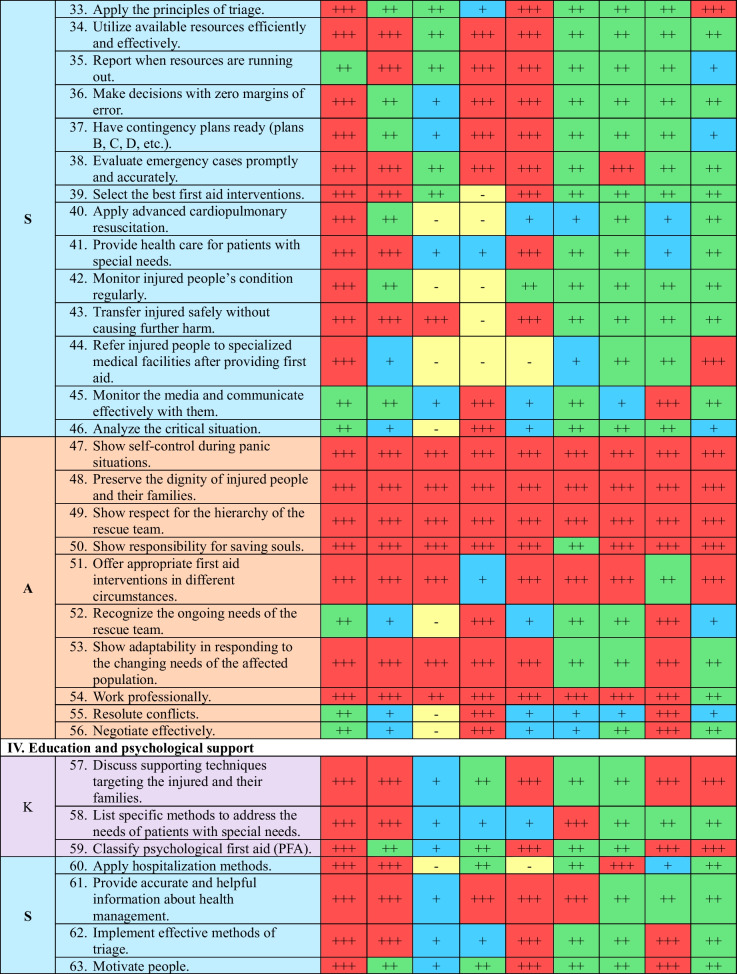

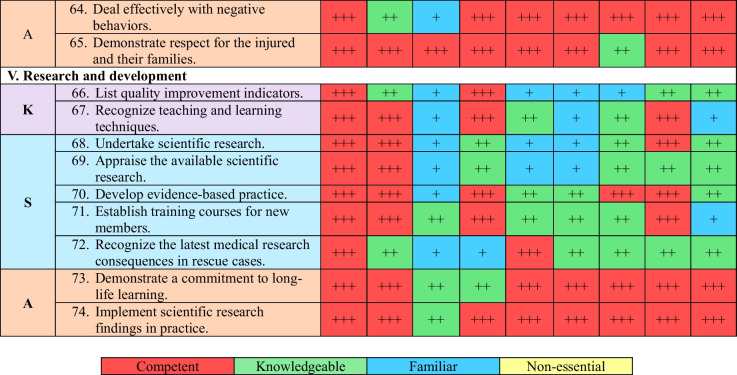
The Final Competency List for Syrian Health Workers in Emergency Time

MD: Medical Doctors, N: Nurses, AHP: Allied Healthcare Professionals, M: Managers, ODT: On-site Disaster Team, Ph: Pharmacists, D: Dentists, PSP: Psychological Support Professionals, MS: Medical Students, K: Knowledge, S: Skills, A: Attitude

The relative importance of competencies can vary among the nine groups of health workers, depending on their specific responsibilities in earthquake response, as outlined in Table 3. Consequently, each group has a unique and essential set of critical competencies that are crucial for their respective roles during earthquake response. The breakdown of competencies is presented in Table 3.

**Table 3:** Allocation of competencies among the nine different groups of Syrian health workers in emergency situations based on their relative importance

Domain/No		MD		N		AHP		M		ODT		PH		D		PSP		MS	
		No	%	No	%	No	%	No	%	No	%	No	%	No	%	No	%	No	%
K/12	C	9	75.0^*^	8	66.7	1	8.3^**^	3	25.0	9	75.0^*^	4	33.3	4	33.3	5	41.7	5	41.7
	Kn	2	16.7	3	25.0	3	25.0	3	25.0	1	8.3	6	50.0	6	50.0	3	25.0	4	33.3
	F	1	8.3	1	8.3	8	66.7	6	50.0	2	16.7	2	16.7	2	16.7	2	16.7	2	16.7
	NE	0	0.0	0	0.0	0	0.0	0	0.0	0	0.0	0	0.0	0	0.0	2	16.7	1	8.3
S/35	C	28	80.0^*^	18	51.4	6	17.1	20	57.1	22	62.9	7	20.0	8	22.9	13	37.1	4	11.4^**^
	Kn	5	14.3	12	34.3	8	22.9	5	14.3	3	8.6	21	60.0	24	68.6	17	48.6	23	65.7
	F	1	2.9	4	11.4	13	37.1	5	14.3	6	17.1	6	17.1	2	5.7	5	14.3	7	20.0
	NE	1	2.9	1	2.9	8	22.9	5	14.3	4	11.4	1	2.9	1	2.9	0	0.0	1	2.9
A/27	C	24	88.9^*^	17	63.0	14	51.9	23	85.2	19	70.4	18	66.7	17	63.0	21	77.8	11	40.7^**^
	Kn	3	11.1	6	22.2	5	18.5	2	7.4	4	14.8	7	25.9	9	33.3	5	18.5	11	40.7
	F	0	0.0	4	14.8	4	14.8	2	7.4	4	14.8	2	7.4	1	3.7	1	3.7	5	18.5
	NE	0	0.0	0	0.0	4	14.8	0	0.0	0	0.0	0	0.0	0	0.0	0	0.0	0	0.0
All/74	C	61	82.4^*^	43	58.1	21	28.4	46	62.2	50	67.6	29	39.2	29	39.2	39	52.7	20	27.0^**^
	Kn	10	13.5	21	28.4	16	21.6	10	13.5	8	10.8	34	45.9	39	52.7	25	33.8	38	51.4
	F	2	2.7	9	12.2	25	33.8	13	17.6	12	16.2	10	13.5	5	6.8	8	10.8	14	18.9
	NE	1	1.4	1	1.4	12	16.2	5	6.8	4	5.4	1	1.4	1	1.4	2	2.7	2	2.7

MD: Medical Doctors (61 competencies), N: Nurses (44 competencies), AHP: Allied Healthcare Professionals (21 competencies), M: Managers (46 competencies), ODT: On-site earthquake Team (48 competencies), Ph: Pharmacists (30 competencies), D: Dentists (29 competencies), PSP: Psychological Support Professionals (40 competencies), MS: Medical Students (20 competencies), K: Knowledge, S: Skills, A: Attitude. Kn: Knowledgeable, C: Competent, F: Familiar, N: Non-essential

*Highest value

**Lowest value

## Discussion

Earthquakes are among the most common natural earthquakes worldwide, causing massive destruction and multiple casualties [27, 28]. Therefore, health workers should be able to respond appropriately when an earthquake occurs. On February 6, 2023, the Eastern Mediterranean region suffered multiple quakes and aftershocks that had never been witnessed since 1939. Medical teams worked tirelessly to provide health care to the injured [29, 30]. However, more work needs to be undertaken to determine the prerequisite competencies of various health workers during earthquakes. This is where the role of competency-based medical education needs to be addressed.

All health workers who provide medical care and psychological support for the injured, at site locations or in hospitals should have the essential competencies to approach patients affected by the disaster [31].

The designed framework, in the current study, operates with a three-component model of competency including knowledge, skills, and attitudes. Since all three (knowledge, skill, and attitude) directly affect the performance of an individual as well as an organization.

The Delphi process aimed to find common competencies to establish a framework for healthcare team leaders to build an integrated team as well as to identify specific competencies for each group.

The classification we have adopted for preparing the team for the rescue process during and after the earthquake can ensure a comprehensive perspective of the rescue process and proper preparation for future disasters. Each category was then subdivided according to the three domains of learning: knowledge (cognitive), skills (psychomotor), and attitudes (affective) [32]. The presence of these abilities together in the health worker will help maintain patients' physical and psychological health during earthquakes.

Furthermore, based on the work of George Miller [32, 33], each competency was evaluated for each role of health workers and adopted a relevant hierarchy as proposed; non-essential, familiar, knowledgeable, and competent. Logically, not all health workers should develop all the competencies identically. For example, in the hospital, doctors must provide skilled professional medical care to the injured. However, dealing with survivor detection equipment is non-essential for them, as it is a critical task of the on-site disaster team.

This study used qualitative methods to derive context-specific skills for Syrian health workers managing earthquakes, identifying 74 competencies with three categories including 12 knowledge items, 35 skills items, and 27 attitude items.

Previous work has engaged multidisciplinary experts to develop 11 core and 36 sub-competencies applicable across various disasters and settings, aiming to advance disaster preparedness internationally. However, researchers have not addressed the value of collaborative, multidisciplinary approaches to effective disaster response [7].

Our study highlighted key aspects of earthquake preparedness education for healthcare workers, including the need for effective, standardized, evidence-based training programs that outline healthcare providers' vital roles.

However, Hsu et al. [12] used an evidence-based consensus approach to develop seven broad competencies for all hospital healthcare workers irrespective of their role. Our work focused on creating role-specific competencies for earthquake response, applying a Delphi technique. Both studies concur on key principles, but differ in their competency development methods and level of specificity based on the targeted healthcare worker population.

This study emphasized the need for prompt development of competencies to enable health workers to effectively manage earthquakes and enhance national disaster resilience. In contrast, the scoping review by Su et al. [14] comprehensively catalogs requisite nursing skills for various crises, serving as a baseline to delineate expected competency levels. While differing in scope, both highlight key disaster response skills like clinical competence, communication, ethics, caring, and addressing physical/mental health needs. This study advocates for urgent competency building to improve disaster management, whereas Su et al. [14] map out fundamental skills as a starting point. Together, they emphasized the importance of both targeted competencies and foundational skills to enhance earthquake and broader disaster response.

The earthquakes in Turkey and Syria have highlighted the need for improving disaster preparedness among health workers, particularly managers and nurses. Strategies should be implemented to enhance nurses' disaster response skills, such as establishing disaster committees that include nurses [5]. Collaborative, multidisciplinary approaches are important for effective earthquake response.

When identifying competencies, it is important to consider various factors. These include meeting the public's requirements and expectations about disasters and emergencies, addressing existing health issues within the community, understanding the distinct characteristics of the population being served, and having a comprehensive understanding of the unique clinical setting.

The initial inquiry aimed to identify individuals engaged in the healthcare-related earthquake response and subsequently assess their competencies based on their specializations, roles, and responsibilities. To ensure optimal resource allocation, minimize overlapping responsibilities, and foster seamless collaboration among health workers [34], we identified nine distinct roles within the medical team were identified. Each role has its own set of responsibilities, and integrating these roles leads to optimal medical care during earthquakes. We also took into account the field experience of our team members who had firsthand experience in providing medical assistance during such catastrophes. Their insights highlighted the importance of effective earthquake management, which goes beyond basic medical knowledge. Strong communication skills, leadership abilities, and the ability to interact with others were identified as crucial. Additionally, social interaction was recognized as vital for the mental and physical well-being of both rescue teams and survivors. The final list of competencies developed in this study aimed to address and bridge any gaps in previous practice during the Syrian crisis and the recent earthquake. These competencies apply to various hospital personnel who share specialization and expertise backgrounds.

Similar to previous work, specialized medical doctors should be the first group of health workers who can provide professional surgical and medical care at hospitals while considering different specializations, various challenges, and diverse cases [35, 36]. Medical doctors also should provide emergency medical treatment to people trapped or injured in collapsed buildings [37, 38]. They are responsible for making critical decisions within tight time constraints, striving for a shallow margin of error [39, 40].

As with previous work, it can be inferred that nursing staff play a crucial role as the second pivotal group in addressing the multifaceted challenges caused by disasters. They provide essential support to medical doctors and offer physical and psychological care to the injured [33, 41]. These group members also assume critical responsibilities in earthquake preparedness and response within hospitals and communities [14, 42]. However, studies indicate that nursing staff is often not adequately prepared to effectively respond to the complex demands of disasters [43–45], primarily due to the inadequate integration of national disaster policy frameworks [46]. Consequently, there is a need to prepare Syrian nursing staff for these catastrophic situations through the implementation of integrated multidisciplinary programs.

Disasters involving mass casualties present a multitude of challenges that can only be effectively managed by well-trained leaders [47]. For this reason, we have identified hospital managers and leaders of rescue teams as a separate category. These events often result in patient surges that overwhelm hospital resources, space, and staff, necessitating leaders to make courageous decisions in the face of limited resources. Influential leaders should anticipate future needs and play critical roles in planning, collaborating, communicating, and preparing for the rescue process [48–50].

Dentists are essential healthcare team members, yet their role has not been emphasized in disasters. It has primarily been confined to the traditional forensic odontology in identifying victims after disasters [51]. However, subsequent experiences have demonstrated the successful utilization of dentists in the disaster emergency medical response system [41, 52, 53]. The findings of the current study highlight the importance of a specific set of competencies that define dentists as vital contributors to medical surge events during active disaster response. This is due to their continuous learning, competency in evidence-based practice, and ability to implement scientific research findings [54, 55].

Pharmacists contribute significantly to disaster response efforts by effectively collaborating with other healthcare providers. Their responsibilities extend beyond dispensing medications under established protocols. Pharmacists also offer counseling and guidance to patients regarding medication usage, addressing any concerns or queries related to treatment and medications [56, 57].

Medical students can assist in mass casualty situations under proper medical supervision when hospitals are overwhelmed. Well-prepared medical students can contribute effectively to disaster response efforts [58–60].

The on-site disaster team constitutes a multidisciplinary team responsible for providing on-scene care. They serve as the first line of defense in managing the increasing number of patients during such disasters. Ensuring the safety of these responders is a priority, necessitating the use of personal protective equipment, measures to mitigate the risk of injury, and comprehensive training to manage stress effectively [61]. Disaster responders should perform triage, as patients with life-threatening injuries require immediate stabilization and evacuation to more advanced healthcare facilities [62].

Psychological support professionals are critical in offering PFA as a vital intervention in providing early psychological assistance to survivors of natural disasters during the initial response [63]. PFA can be administered by mental health workers and 'peer responders' such as volunteers, first responders, and even public members [64]. PFA aims to promote safety, stabilize disaster survivors, and connect them with necessary support and resources [65]. Psychological support providers are also responsible for conducting mental health and psychosocial needs assessments, organizing healing activities for children, offering support to staff and volunteers, and providing essential psychological information to the affected population [66].

Allied healthcare professionals and other stakeholders, including volunteers, non-governmental organization staff, and civil defense members, can effectively assist the medical team if they are well-prepared [6, 67].

The literature shows a previous qualitative study [33] conducted in Iran to identify core competencies for nurses during the Kermanshah earthquake in 2017. Similar to our results, the study concluded that several essential competencies are classified into four groups: clinical competency (cardiopulmonary resuscitation skills and airways management), personal competencies (communication skills, resiliency, and creativity in providing care), ethical competencies (commitment to ethics and professional responsibility), and fundamental caring skills (triage, psychological care, and monitoring skills) [68]. Despite differences in grouping and categories, our study shares similar competencies.

Our study is in agreement with another undertaken by a multidisciplinary working group representing clinical medicine, nursing, public health, adult education, and emergency management, which identified 11 core competencies and 36 sub-competencies required for health workers during disasters. These competencies provide a useful starting point for delineating the expected competency levels of health workers in disaster medicine and public health [7]. However, it should be emphasized that this study is unique in that the burden and responsibilities of Syrian health workers are increased to respond effectively to the earthquake due to a compromised health system that has been severely damaged due to more than a decade of the Syrian crisis. The lack of medical resources due to long-lasting sanctions [68], destruction of health facilities, and shortage of healthcare providers [69]. In addition, up to 50% of the health facilities have been destroyed, including Al-Kindy Hospital, the largest hospital in the Middle East, located in northern Syria, which was destroyed during the armed conflict in late 2013 [69]. The scarcity of health workers is a pressing issue in Syria as many have left the country searching for better opportunities [70, 71], leading to an exodus of almost 70% of medical personnel [69]. Consequently, there is a chronic shortage of healthcare personnel and an increased workload on the remaining medical staff [69]. To address this chronic challenge, serious steps should be undertaken to enhance the healthcare system in Syria and increase the preparedness of medical teams to face earthquakes.

In this study, the selection of participants, with several perspectives, opinions, and multidisciplinary backgrounds, has enabled us to identify members who should appropriately respond during the earthquake and to provide a comprehensive list of competencies required for providing the best care and medical support to affected people. However, for the sake of completeness, future work should take into consideration involving patients and their families, who experienced earthquakes. This is significant because these individuals have a unique understanding of earthquake challenges and needs. As a result, their input can provide valuable insights into the competencies needed by health workers in such situations.

It should be addressed that there is no right number of participants in a Delphi panel in the medical education literature [72]. In the current study, we emphasized the diversity of perspectives and opinions by multidisciplinary selections. However, the number of participants in the current study was small N = 9 and this can be a limitation. Future work should further test the identified competencies in this study and should consider competencies for other natural disasters.

It is very important to point out that integrating those competencies into medical curricula would greatly enhance their implementation by health workers in the future and prepare them for such catastrophic events.

At present, the acquisition of these competencies and the operationalization of the competency framework can be performed by developing international strategies to design collaborative regular training programs that can be implemented by local authorities, community, or international organizations to support health workers in acquiring these competencies within a very strained health system.

## Conclusion

The competencies of health workers should be tailored to the specific needs of the affected communities for an effective response.

Responding to earthquakes needs more than medical knowledge and skills. A list of core competencies was developed to address the diverse health needs of populations impacted by the Syrian earthquake. It is hoped that this list will enhance a country’s resilience and will enable decision –makers to equip health workers with these competencies and prepare them for similar situations in Syria and other countries.

## Data Availability

The data can be available upon request from the corresponding author.

## Abbreviations

MEP: Medical Education Program
PFA: Psychological First Aid
SVU: Syrian Virtual University
